# The Cpx envelope stress response both facilitates and inhibits elaboration of the enteropathogenic *Escherichia coli* bundle-forming pilus

**DOI:** 10.1111/j.1365-2958.2010.07145.x

**Published:** 2010-04-29

**Authors:** Stefanie L Vogt, Anna Z Nevesinjac, Romney M Humphries, Michael S Donnenberg, Glen D Armstrong, Tracy L Raivio

**Affiliations:** 1 Department of Biological Sciences, University of AlbertaEdmonton, Alberta, Canada; 2 Department of Microbiology and Infectious Diseases, University of CalgaryCalgary, Alberta, Canada; 3 Division of Infectious Diseases, University of Maryland School of MedicineBaltimore, MD, USA

## Abstract

The Cpx envelope stress response is induced by the misfolding of periplasmic proteins and restores envelope homeostasis by upregulating several periplasmic protein folding and degrading factors. The Cpx response also regulates the expression of a variety of envelope-spanning protein complexes, including flagella, secretion systems and pili, which play an important role in pathogenesis. In a previous study, we inactivated the Cpx response in enteropathogenic *Escherichia coli* (EPEC), a causative agent of infant diarrhoea, and observed decreased expression of its major adhesin, the bundle-forming pilus (BFP). Here, we examined the mechanism underlying this BFP expression defect, and found that this phenotype can be attributed to insufficient expression of periplasmic folding factors, such as DsbA, DegP and CpxP. Hence, a low level of Cpx pathway activity promotes BFP synthesis by upregulating factors important for folding of BFP component proteins. Conversely, we found that full induction of the Cpx response inhibits BFP expression, mainly by repressing transcription of the *bfp* gene cluster. In combination with a previous report examining EPEC type III secretion, our results demonstrate that the Cpx response co-ordinates the repression of cell-surface structures during periods of envelope stress.

## Introduction

The Gram-negative bacterial envelope is the cell's first point of contact with the external environment, and also contains many structures crucial for survival in diverse habitats. Organisms such as *Escherichia coli* have therefore evolved numerous regulatory systems capable of detecting and responding effectively to envelope stress. One such system is the Cpx signal transduction pathway, which is composed of the inner membrane (IM) histidine kinase CpxA and the cytoplasmic response regulator CpxR. In accordance with the behaviour of other two-component systems, CpxA autophosphorylates upon detecting an inducing cue (Raivio and Silhavy, 1997). Subsequently, CpxA phosphorylates CpxR, thereby enabling the response regulator to bind to DNA and modify gene transcription (Raivio and Silhavy, 1997). In the absence of envelope stress, CpxR is maintained in an inactive state by the phosphatase activity of CpxA (Raivio and Silhavy, 1997). One unorthodox aspect of the Cpx system is that a third component, a small periplasmic protein called CpxP, inhibits Cpx pathway activation under non-inducing conditions (Raivio *et al*., 1999). Since mutation of the CpxA periplasmic sensing domain results in the loss of CpxP-mediated inhibition, it is believed that this inhibition occurs through a direct interaction between the two proteins (Raivio *et al*., 1999).

Although the precise molecular nature of the envelope perturbation that activates the Cpx response has not been determined, a number of inducing cues have been identified. Known activators of the Cpx response include alkaline pH (Danese and Silhavy, 1998), membrane composition alterations (Mileykovskaya and Dowhan, 1997; Danese *et al*., 1998) and overproduction of pilus component proteins such as PapE, PapG and BfpA (Jones *et al*., 1997; Nevesinjac and Raivio, 2005). Since all of these inducing cues are expected to generate misfolded envelope proteins, one potential physiological role of the Cpx pathway is to monitor periplasmic protein folding (MacRitchie *et al*., 2008a). An additional Cpx-activating signal is the overexpression of the outer membrane (OM) lipoprotein NlpE (Snyder *et al*., 1995). NlpE is required for induction of the Cpx response when *E. coli* cells adhere to hydrophobic surfaces (Otto and Silhavy, 2002), but does not play a role in Cpx sensing of stresses such as alkaline pH or overexpression of Pap pilus subunits (DiGiuseppe and Silhavy, 2003). Therefore, the Cpx pathway may detect bacterial adhesion to surfaces in addition to sensing envelope stress.

Once the Cpx response has been activated, CpxR upregulates the expression of numerous genes whose products are involved in envelope protein folding, ameliorating the envelope stress. One of the first identified Cpx regulon members is the periplasmic enzyme DegP (Danese *et al*., 1995), which possesses both protease and chaperone functions and therefore is important for envelope protein quality control (Spiess *et al*., 1999). Other Cpx-regulated proteins include DsbA, the primary disulphide bond oxidoreductase in the *E. coli* periplasm (Danese and Silhavy, 1997; Pogliano *et al*., 1997; Heras *et al*., 2009) and PpiA (also known as RotA), which catalyses *cis-trans* peptide bond isomerization around proline residues in periplasmic proteins (Pogliano *et al*., 1997). Phosphorylated CpxR also activates transcription of the *cpxRA* and *cpxP* operons, thereby endowing the system with both positive and negative feedback mechanisms (Danese and Silhavy, 1998; Raivio *et al*., 1999). Interestingly, the Cpx pathway inhibitor CpxP also plays a stress-combative role, by facilitating degradation of misfolded P-pilus proteins by DegP (Isaac *et al*., 2005).

Phosphorylated CpxR is also capable of repressing target genes, many of which encode bacterial cell surface appendages. Included in this category are the flagellar motor, chemotaxis and aerotaxis genes *motAB-cheAW*, *tsr* and *aer* (De Wulf *et al*., 1999; 2002; Price and Raivio, 2009); the *csgBA* and *csgDEFG* operons, encoding components of the curli fimbriae and its transcriptional regulator (Dorel *et al*., 1999; Prigent-Combaret *et al*., 2001); and the *pap* genes encoding the uropathogenic *E. coli* P-pilus (Hernday *et al*., 2004). The Cpx-mediated repression of these genes encoding envelope-localized structures may reflect the benefit of reducing non-essential envelope protein traffic during periods of envelope stress (MacRitchie *et al*., 2008a). Recent evidence indicates that the Cpx pathway is frequently involved in regulating structures required for virulence in pathogenic Gram-negative bacteria (reviewed by Raivio, 2005), including enteropathogenic *E. coli* (EPEC).

Enteropathogenic *E. coli* is a common cause of acute diarrhoea among infants and young children in developing countries, and has been associated with occasional disease outbreaks in daycares and hospitals in industrialized nations (Chen and Frankel, 2005). EPEC pathogenesis is thought to proceed by three major steps: (i) initial adherence to epithelial cells of the small intestine, (ii) signal transduction via a type III secretion system (T3SS), and (iii) intimate adherence, which is associated with enterocyte effacement and the formation of a pedestal beneath the bacterial cell (Chen and Frankel, 2005). Several adhesins, including flagella, EspA filaments and the *E. coli* common pilus (Cleary *et al*., 2004; Saldaña *et al*., 2009), have been proposed to play a role in the initial attachment of EPEC to epithelial cells. However, in typical EPEC strains, the bundle-forming pilus (BFP) is likely the predominant adhesin (Cleary *et al*., 2004). The primary BFP pilin, bundlin, binds to *N*-acetyllactosamine-like receptors on human cells (Hyland *et al*., 2008), and BFP filaments from adjacent bacterial cells can also intertwine in rope-like bundles (Girón *et al*., 1991). These properties of BFP give rise to EPEC's characteristic localized adherence (LA) phenotype, where bacteria adhere to tissue culture cells in discrete microcolonies (Scaletsky *et al*., 1984).

As type IV pili, BFP comprise a complex of proteins that spans all cellular compartments. The extracellular pilus filament contains mainly, and possibly only, polymerized bundlin residues. The pilus is extruded through a donut-like complex of the secretin BfpB, a major component of the OM subassembly of the BFP (Schmidt *et al*., 2001; Ramer *et al*., 2002; Daniel *et al*., 2006). The periplasmic component BfpU performs an unknown, yet essential, role in BFP elaboration (Schreiber *et al*., 2002). The pilus filament is anchored to the IM by a scaffold composed of the polytopic protein BfpE (Ramer *et al*., 2002), which interacts with the additional IM component BfpC (Crowther *et al*., 2004). Finally, two cytoplasmic ATPases are associated with the pilus: BfpD provides the energy required for extension of the pilus filament (Anantha *et al*., 2000), while BfpF permits retraction of the BFP (Anantha *et al*., 1998). BfpF may enhance bacterial transmission by permitting disaggregation of individual bacterial cells from microcolonies, allowing the released cells to colonize other areas of the intestine or to be shed by the host (Bieber *et al*., 1998; Knutton *et al*., 1999).

All of the genes encoding BFP components are located in a single 14-gene cluster on the large EPEC attachment factor (EAF) plasmid (Sohel *et al*., 1996; Stone *et al*., 1996). Non-pathogenic *E. coli* K-12 strains are capable of elaborating BFP when this *bfp* gene cluster is expressed from an inducible promoter (Stone *et al*., 1996). The native *bfp* gene cluster in EPEC is highly regulated at the transcriptional level. *bfpA* transcription is increased by the presence of calcium ions and decreased by ammonium ions; transcription is maximal during exponential phase and at a temperature of 37°C (Puente *et al*., 1996). Moreover, *bfpA* expression requires the transcriptional activator PerA (BfpT), which is also encoded on the EAF plasmid (Tobe *et al*., 1996). Regulation of *perA* expression is similarly complex, being enhanced by the EPEC quorum sensing cascade (Sperandio *et al*., 1999; Sircili *et al*., 2004), the Pst phosphate-specific transport system (Ferreira and Spira, 2008), and PerA binding to its own promoter (Martínez-Laguna *et al*., 1999). *perA* transcription is repressed by ammonium ions, temperatures above or below 37°C, and the acid resistance regulator GadX (Martínez-Laguna *et al*., 1999; Shin *et al*., 2001). These observations suggest that PerA assimilates various environmental signals in order to ensure that BFP are elaborated only under favourable conditions.

Recently, our laboratory has begun to examine the contribution of the Cpx pathway to EPEC virulence gene regulation. MacRitchie *et al*. (2008b) demonstrated that mutational inactivation of the Cpx pathway has little effect on EPEC T3S; however, activation of the pathway inhibits T3S, at least in part by repressing transcription of translocator and effector genes. Furthermore, we observed that an EPEC *cpxR* mutant has reduced expression of bundlin compared with wild-type EPEC and is defective in the BFP-mediated process of LA (Nevesinjac and Raivio, 2005). In the current study, we examined the basis for the decreased bundlin expression of *cpxR* null EPEC. Although we found that transcription of *bfpA* was not significantly altered in this strain, we determined that several Cpx-regulated folding factors are required for proper BFP biogenesis, suggesting that the *cpxR* mutant has a reduced ability to properly fold BFP protein components. In light of the seemingly conflicting observations that the Cpx pathway positively influences BFP biogenesis but negatively regulates T3S, we also investigated the effect of Cpx pathway activation upon the BFP. We found that, similarly to the T3SS, BFP expression is repressed at the transcriptional level during the Cpx response, demonstrating for the first time that the Cpx response can mediate either positive or negative effects upon a single cell-surface structure, depending on the level of pathway activity.

## Results

### Cpx-regulated periplasmic protein folding and degrading factors are required for normal BFP biogenesis

One potential explanation for the reduced BFP expression of an EPEC *cpxR* null mutant (Nevesinjac and Raivio, 2005) is that this strain produces insufficient amounts of periplasmic protein folding factors to ensure proper folding of BFP component proteins. This hypothesis seemed particularly plausible given that Zhang and Donnenberg (1996) previously demonstrated that DsbA is required for stability of the BFP major pilin, bundlin. To examine the role of Cpx-regulated protein folding and degrading factors in BFP biogenesis, we constructed EPEC mutants with null alleles of the genes *dsbA*, *degP*, *cpxP* and *ppiA*, all of which are positively regulated by the Cpx response in EPEC as well as in *E. coli* K-12 (D.M. MacRitchie *et al*., in preparation).

**Fig. 1:**
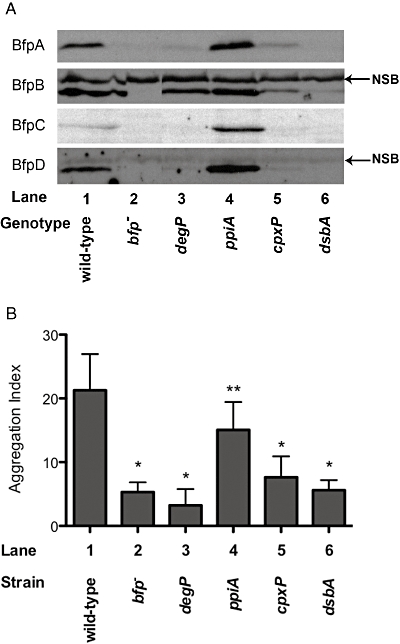
BFP expression is reduced in EPEC mutants lacking *dsbA*, *degP* and *cpxP*. A. Western analysis of bundlin (BfpA), BfpB, BfpC and BfpD expression in wild-type and mutant EPEC strains: lane 1, wild-type (E2348/69); lane 2, *bfp^-^* strain (JPN15); lane 3, *degP*::kan (ALN188); lane 4, *ppiA*::kan (ALN190); lane 5, *cpxP*::kan (ALN194); lane 6, *dsbA*::kan (TR1121). Whole-cell lysates were collected from EPEC grown in DMEM/F12 as described in *Experimental procedures*. Samples were collected from each strain at least three times; one representative blot is shown. Arrows denote non-specific bands (NSB). B. Results of autoaggregation assay performed on EPEC Cpx regulon mutants. Autoaggregation assays were performed as described in *Experimental procedures*; the overall average and standard deviation resulting from two separate experiments performed in triplicate are shown. One asterisk (*) denotes a value significantly different from positive control E2348/69; two asterisks (**) denote a value significantly different from both E2348/69 and negative control JPN15 (one-way anova with Bonferroni's multiple comparison test; *P* < 0.05).

To analyse BFP protein levels in the Cpx regulon mutants, whole-cell lysates were collected from wild-type and mutant EPEC strains grown under conditions favourable for BFP expression (see *Experimental procedures*). Western blotting revealed that the expression of bundlin is eliminated in the *dsbA* mutant, as expected; bundlin levels are also reduced in the *degP* and *cpxP* mutants (Fig. 1A). Mutation of *ppiA*, on the other hand, did not reduce bundlin levels (Fig. 1A). Interestingly, the *dsbA*, *degP* and *cpxP* mutations affected not only the abundance of the pilin monomer, but also the expression of other BFP proteins spanning multiple cellular compartments (Fig. 1A). Production of the OM secretin BfpB, the IM component BfpC and the cytoplasmic ATPase BfpD was reduced in these strains, suggesting that not just the pilin protein but the entire BFP apparatus is influenced by the activity of the Cpx pathway. Expression of the BFP proteins could be restored in the *degP*, *cpxP* and *dsbA* mutants by complementation *in trans* (data not shown).

Since the expression of bundlin was reduced but not abolished in several Cpx regulon mutants, we examined the functionality of the BFP expressed by these EPEC strains using autoaggregation and LA assays. The autoaggregation assay measures the ability of EPEC strains to form aggregates when grown in liquid culture, a phenotype that is correlated with BFP elaboration (Anantha *et al*., 1998). All of the Cpx regulon mutants, including strains lacking *degP*, *ppiA*, *cpxP* and *dsbA*, had a reduced capacity to aggregate under these assay conditions compared with the wild-type strain E2348/69 (Fig. 1B). Among these strains, only the *ppiA* mutant was able to aggregate significantly better than the *bfp*-negative control strain JPN15, in accordance with the higher levels of BFP protein synthesis in this strain (Fig. 1A).

Localized adherence assays were performed to assess whether the Cpx regulon mutants were compromised in their ability to adhere to host cells. The *dsbA* mutant was not examined for LA, since it was previously demonstrated that LA is abolished in the absence of DsbA (Zhang and Donnenberg, 1996). After 1 h of interaction between bacteria and host cells, the *degP* mutant had formed significantly fewer microcolonies than the wild-type control E2348/69, adhering to only 33.4% as many host cells as did the wild-type strain (*P* < 0.0001, Fisher's exact test). The *cpxP* mutant was slightly but reproducibly impaired in LA as well, adhering to 84.9% as many host cells as E2348/69 (*P* < 0.0001, Fisher's exact test). The *ppiA* mutant, however, was able to adhere at wild-type levels (101% of control, *P* > 0.05).

**Fig. 2:**
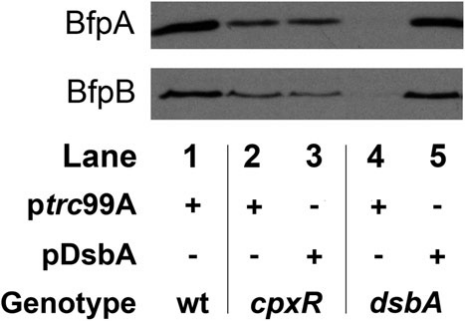
Overexpression of *dsbA* in an EPEC cpxR mutant does not restore BFP synthesis. Western blotting was used to detect expression of the proteins bundlin (BfpA) and BfpB in the wild-type strain E2348/69 (p*trc*99A) (lane 1), the *cpxR* mutant ALN88 (p*trc*99A) (lane 2), ALN88 (pDsbA) (lane 3), the *dsbA* mutant TR1121 (p*trc*99A) (lane 4) and TR1121 (pDsbA) (lane 5). Whole-cell lysates were collected from EPEC grown in DMEM/F12 without IPTG as described in *Experimental procedures*. Samples were collected from each strain at least three times; one representative blot is shown.

Mutation of *dsbA* clearly had the largest influence on BFP protein levels (Fig. 1A) and LA (Zhang and Donnenberg, 1996) among the Cpx regulon members tested. It was therefore possible that the BFP expression defect of the *cpxR* mutant might be entirely attributable to decreased expression of *dsbA*. To test this hypothesis, we overexpressed *dsbA* from an inducible promoter in both the *dsbA* and *cpxR* mutants. Western blotting to detect two BFP components (bundlin and BfpB) revealed that, although pDsbA substantially increased BFP protein synthesis in the *dsbA* mutant, the overexpression of *dsbA* did not increase BFP expression in the *cpxR* mutant (Fig. 2). The BFP expression defect of the *cpxR* mutant therefore cannot be entirely explained by its decreased DsbA expression.

Overall, these results demonstrate that Cpx-regulated periplasmic protein folding and degrading factors, including DsbA, DegP, CpxP and possibly others yet to be identified, are required for normal elaboration of the EPEC BFP.

### Transcription of *bfpA* is not significantly affected by mutation of *cpxR*

Since phosphorylated CpxR is capable of modulating transcription of target genes, we wished to assess whether transcription of the *bfp* operon or the gene encoding its transcriptional regulator, *perA*, might also be altered in the EPEC *cpxR* mutant. To measure transcription, we constructed *bfpA–lux* and *perA–lux* transcriptional fusions using the previously described reporter plasmid pJW15 (MacRitchie *et al*., 2008b). These fusions were designed to contain all of the upstream regulatory elements known to be important for regulation of *bfpA* and *perA* transcription (Puente *et al*., 1996; Martínez-Laguna *et al*., 1999). We validated the use of these fusions to measure *bfpA* and *perA* transcription by comparing their activity under different culture and strain conditions to the activity of previously published *bfpA-cat* and *perA-cat* fusions (Puente *et al*., 1996; Martínez-Laguna *et al*., 1999). Both the *bfpA–lux* and *perA–lux* reporters were (i) expressed at higher levels in Dulbecco's modified Eagle's medium (DMEM) than in Luria–Bertani (LB), (ii) expressed at considerably lower levels in EPEC lacking *perA* than in wild-type EPEC, and (iii) repressed by the addition of 20 mM ammonium sulphate to the DMEM growth medium (data not shown).

**Fig. 3:**
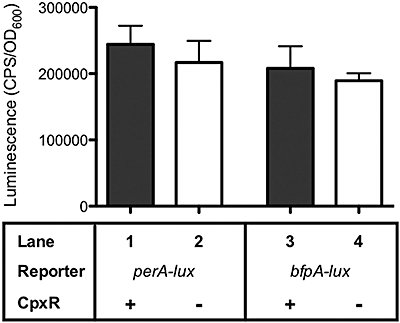
Transcription of *bfpA* and *perA* is not affected by a *cpxR* null mutation. After overnight growth in LB, wild-type and *cpxR* mutant EPEC strains transformed with the *bfpA–lux* and *perA–lux* reporter plasmids were subcultured 1:100 into DMEM/F12 as described in *Experimental procedures*. Luminescence (cps, counts per second) and optical density of the culture (OD_600_) were measured every 2 h. The normalized luminescence was calculated by subtracting the luminescence reading of a medium blank from the luminescence of the culture sample, then dividing that value by the OD_600_ of the culture, reduced by the OD_600_ of the blank. Experiments were performed in quintuplicate at least twice; the mean and standard deviation from the 4 h reading from one experiment are shown.

To assess the effect of a *cpxR* null mutation upon *bfpA* and *perA* transcription, each reporter was transformed into EPEC wild-type and *cpxR* mutant strains. Bacteria were subcultured into DMEM, and the activity of the reporters was monitored every 2 h up to 8 h post subculture. No consistent difference in the activity of either reporter could be detected between wild-type and *cpxR* null strains at any time point (*P* > 0.05, unpaired *t*-tests) (Fig. 3). In conjunction with the results described above, these data suggest that reduced transcription of the *bfp* gene cluster cannot explain decreased BFP elaboration in the EPEC *cpxR* mutant, while a diminished level of folding factors, such as DegP, CpxP and DsbA, could account for this difference.

### Activation of the Cpx response inhibits BFP elaboration

**Fig. 4:**
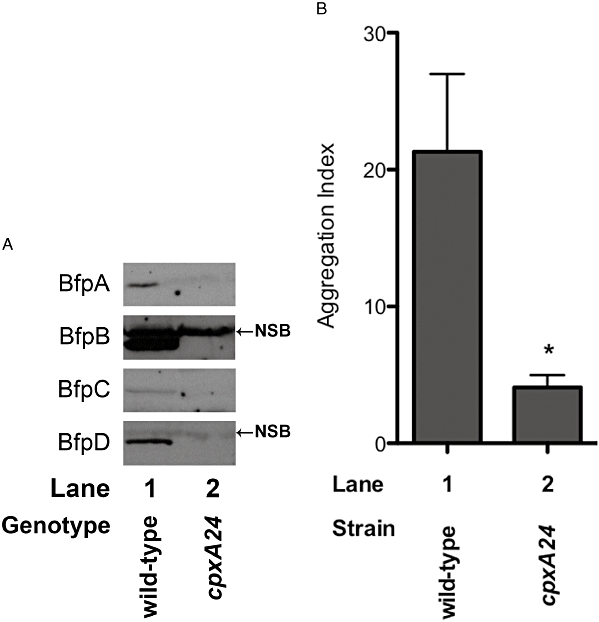
BFP elaboration is inhibited by the *cpxA24* gain-of-function mutation. A. Western analysis of bundlin (BfpA), BfpB, BfpC and BfpD expression in wild-type strain E2348/69 (lane 1) and *cpxA24* mutant EPEC strain ALN195 (lane 2). Whole-cell lysates were collected from EPEC grown in DMEM/F12 as described in *Experimental procedures*. Samples were collected from each strain at least three times; one representative blot is shown. Arrows denote non-specific bands (NSB). B. Results of autoaggregation assay performed on wild-type (lane 1) and *cpxA24* mutant (lane 2) EPEC. Autoaggregation assays were performed as described in *Experimental procedures*; the overall average and standard deviation resulting from two separate experiments performed in triplicate are shown. The asterisk (*) denotes a value significantly different from positive control E2348/69 (*P* < 0.0001, unpaired *t*-test).

In order to determine whether Cpx pathway activation would reduce BFP expression as is the case with EPEC T3S, we compared BFP synthesis in wild-type EPEC with that of an EPEC *cpxA24* mutant (MacRitchie *et al*., 2008b). The *cpxA24* mutation, arising from a deletion of approximately 30 amino acids in the periplasmic sensing domain of CpxA, results in constitutive activation of the Cpx response regardless of the presence or absence of inducing cues (Raivio and Silhavy, 1997). Strikingly, the BFP component proteins bundlin, BfpB, BfpC and BfpD were undetectable in the *cpxA24* mutant EPEC (Fig. 4A). Further experiments revealed that this strain exhibited significantly reduced autoaggregation (*P* < 0.0001, unpaired *t*-test) (Fig. 4B); as expected based on the Western blot results, the ability of the *cpxA24* mutant to aggregate was comparable to that of the *bfp^-^* control strain JPN15 (not shown). Furthermore, the *cpxA24* mutant was incapable of forming microcolonies on epithelial cells during the LA assay, while the wild-type strain E2348/69 formed microcolonies on 83.5% of epithelial cells under the conditions used in this assay. These results demonstrate that the *cpxA24* constitutively active mutation dramatically inhibits BFP expression.

**Fig. 5:**
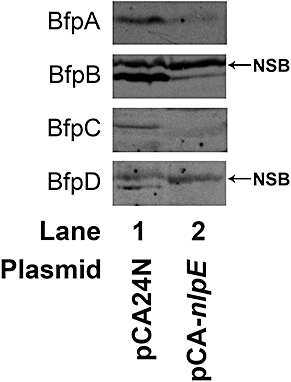
BFP expression is reduced when the Cpx response is activated by overexpressing NlpE. Western analysis of bundlin (BfpA), BfpB, BfpC and BfpD expression in wild-type strain E2348/69 harbouring the vector control pCA24N (lane 1) or the overexpression plasmid pCA-*nlpE* (lane 2). Whole-cell lysates were collected from EPEC grown in DMEM/F12 as described in *Experimental procedures*. Subcultures were grown for 2 h before being induced with 1 mM IPTG, followed by an additional 3 h incubation at 37°C. Samples were collected from each strain at least three times; one representative blot is shown. Arrows denote non-specific bands (NSB).

To confirm that the inhibition of BFP synthesis could be reproduced with different methods of Cpx pathway activation, we also examined the effect of overexpressing the lipoprotein NlpE. This inducing cue has the advantage of being relatively specific to the Cpx pathway, unlike more general cues such as alkaline pH, and also allows us to activate the wild-type Cpx two-component system. Compared with the vector control, wild-type EPEC overexpressing NlpE produced reduced amounts of the BFP components bundlin, BfpB, BfpC and BfpD (Fig. 5). Although NlpE overexpression resulted in a less pronounced phenotype than mutation of *cpxA*, these results confirmed that activation of the Cpx response inhibits BFP elaboration.

### Cpx pathway activation primarily affects BFP at the transcriptional level

**Fig. 6:**
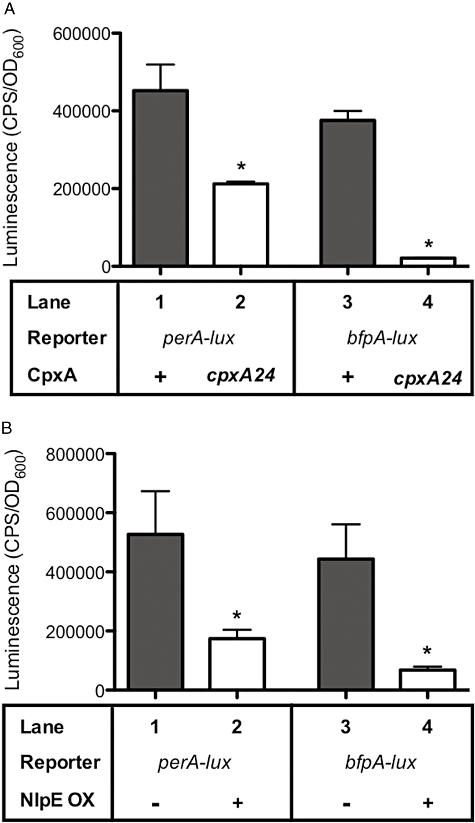
Transcription of *bfpA* and *perA* is repressed during the Cpx response. EPEC strains harbouring *bfpA–lux* and *perA–lux* reporters were subcultured in DMEM/F12 as described in *Experimental procedures*. Normalized luminescence, in units of counts per second (cps) per optical density of the culture (OD_600_) was measured hourly and calculated as described in the Fig. 3 caption. Experiments were performed in quintuplicate at least twice; the mean and standard deviation from one representative experiment are shown. An asterisk (*) denotes significant difference from the wild-type or vector control (*P* < 0.05, unpaired *t*-test). A. Comparison of *bfpA–lux* and *perA–lux* activity in wild-type strain E2348/69 and *cpxA24* mutant ALN195. Results presented are from 4 h post subculture. B. Comparison of *bfpA–lux* and *perA–lux* activity in wild-type EPEC harbouring the vector control pCA24N or the overexpression plasmid pCA-*nlpE*. Strains were subcultured into DMEM/F12 for 2 h, then induced with 0.1 mM IPTG. Results presented are from 5 h post induction.

To begin to uncover the mechanism of BFP inhibition in the *cpxA24* mutant, we examined expression of the *bfpA–lux* and *perA–lux* transcriptional reporters in this strain compared with wild-type EPEC. The activity of both reporters was reduced in the *cpxA24* strain (Fig. 6A). The activity of the *bfpA–lux* reporter was reduced about 20-fold relative to the wild-type strain, and this reduction was consistent regardless of the growth phase of the cultures (data not shown). The *perA–lux* reporter was also expressed at lower levels in the *cpxA24* mutant, but the reduction was milder (only about twofold at the time point shown in Fig. 6A), and was not observed after 6 h or longer post subculture (data not shown). In agreement with the Western blotting results (Figs 4 and 5), overexpression of NlpE also reduced *perA–lux* and *bfpA–lux* activity (Fig. 6B).

**Fig. 7:**
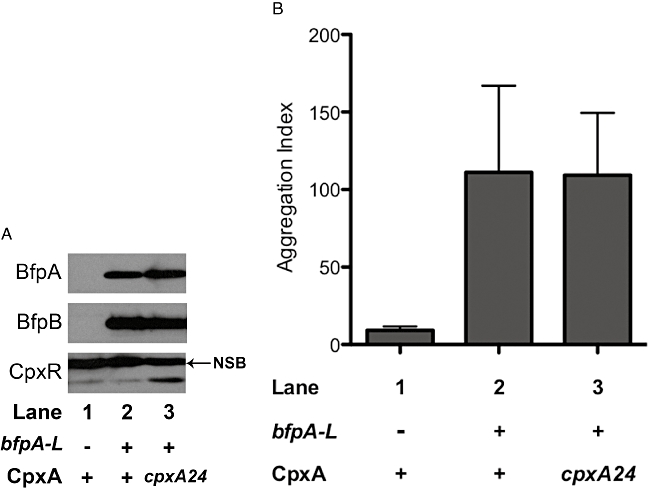
The *cpxA24* mutation does not affect BFP elaboration when the *bfp* genes are expressed from an inducible promoter. A. Western analysis of bundlin (BfpA), BfpB and CpxR expression in the negative control JPN15 (p*trc*99A) (lane 1), the positive control JPN15 (pKDS302), which expresses the *bfpA-L* operon (lane 2), and its isogenic *cpxA24* mutant SV76 (lane 3). Whole-cell lysates were collected from EPEC grown in LB containing 1 mM IPTG as described in *Experimental procedures*. Samples were collected from each strain at least two times; one representative blot is shown. Arrows denote non-specific bands (NSB). B. Results of autoaggregation assay performed on JPN15 (p*trc*99A) (lane 1), JPN15 (pKDS302) (lane 2) and SV76 (lane 3). Autoaggregation assays were performed as described in *Experimental procedures*; the overall average and standard deviation resulting from three separate experiments performed in triplicate are shown.

The strongly decreased transcription of *perA* and *bfpA* in the *cpxA24* mutant suggested that the defect in BFP expression in this strain may be entirely the result of reduced transcription of the *bfp* operon. To separate transcriptional and post-transcriptional effects of the Cpx pathway, we generated a pair of strains in which the *bfp* gene cluster is expressed from a promoter that is not regulated by the Cpx pathway. This was accomplished by transforming the plasmid pKDS302, containing the entire *bfp* gene cluster under the control of an IPTG-inducible promoter (Stone *et al*., 1996), into the pEAF plasmid-cured strain JPN15. Although pEAF contains a total of 115 protein-coding genes (Brinkley *et al*., 2006), no genes other than the *bfp* gene cluster and the *per* operon are known or suspected to have any role in either BFP expression or the Cpx response. This information, along with the ability of JPN15 (pKDS302) to express functional BFP (see Fig. 7), validates the use of this strain in further experiments.

The *cpxA24* mutation was introduced into JPN15 (pKDS302) to give rise to a strain in which the activated Cpx pathway cannot influence BFP expression at the transcriptional level. Western blotting revealed that in this strain background, the *cpxA24* mutation no longer diminished expression of bundlin and BfpB (Fig. 7A). Activation of the Cpx response in this strain was verified by an increased ability to grow on media containing amikacin (data not shown) and an increased cellular level of CpxR (Fig. 7A), resulting from autoactivation of *cpxRA* gene transcription (Raivio *et al*., 1999). Moreover, the autoaggregation assay demonstrated that both the wild-type and *cpxA24* mutant JPN15 (pKDS302) strains are equally capable of expressing functional BFP on the cell surface (Fig. 7B). These results indicate that there is little or no inhibition of BFP expression at the post-transcriptional level in the *cpxA24* mutant.

**Fig. 8:**
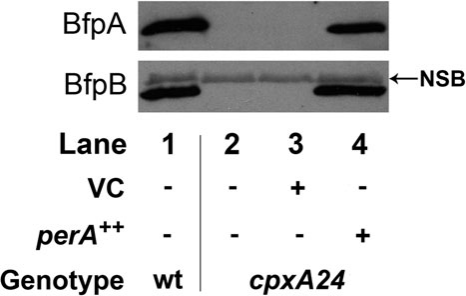
Overexpression of *perA* in an EPEC *cpxA24* mutant restores BFP synthesis. Western blotting was used to detect expression of the proteins bundlin (BfpA) and BfpB in the wild-type strain E2348/69 (lane 1), the *cpxA24* mutant ALN195 (lane 2), the vector control (VC) strain ALN195 (pMPM-K3) (lane 3) and the *perA*-overexpressing strain ALN195 (pCS-A) (lane 4). Whole-cell lysates were collected from EPEC grown in DMEM/F12 with 50 µM IPTG as described in *Experimental procedures*. Samples were collected from each strain at least twice; one representative blot is shown. The arrow denotes a non-specific band (NSB).

Since the defect in BFP expression in the *cpxA24* mutant seemed to be mainly due to decreased transcription, and because expression of *perA* in this strain was repressed, we wished to determine whether BFP expression could be restored to the *cpxA24* mutant by expression of *perA* from an inducible promoter. The *perA* overexpression plasmid pCS-A (Martínez-Laguna *et al*., 1999) was transformed into the *cpxA24* mutant, and BFP expression was monitored by Western blotting. Although bundlin and BfpB could not be detected in the *cpxA24* mutant carrying the vector control plasmid, expression of these proteins was restored to near-wild-type levels in the *cpxA24* mutant overexpressing *perA* (Fig. 8). The ability of a transcriptional activator to restore BFP expression in the *cpxA24* mutant further supports the notion that the defect in BFP elaboration in this strain can largely be attributed to decreased transcription from the *bfpA* promoter.

### Phenotypes of *bfpF cpx* double mutants

Experiments conducted thus far suggested that the *cpxA24* mutation leads to a drastic decrease in pilus assembly, mainly as a result of decreased *bfp* gene transcription, while the *cpxR* mutation does not affect *bfp* transcription, but rather the ability to synthesize a functional pilus. To corroborate these observations, we examined the effect of *cpx* mutations in *bfpF* mutant EPEC, which lacks the ATPase that powers retraction of the BFP (Anantha *et al*., 1998). Studies with type IV pilus retraction double mutant strains have previously been used in other species to separate mutations that prevent pilus assembly from those that simply destabilize the pilus and therefore cause pili to be rapidly retracted (Burrows, 2005). When the first category of mutation is introduced into a pilus retraction mutant, functional pili still cannot be synthesized. However, when the pilus-destabilizing class of mutation is introduced into a pilus retraction mutant, piliation is restored, since even structurally abnormal pili are trapped on the cell surface. We therefore hypothesized that introducing the *cpxA24* mutation into the *bfpF* mutant would strongly reduce BFP elaboration, much like in wild-type EPEC, since *bfp* gene expression, and therefore BFP assembly should be blocked. In contrast, we predicted that introducing a *cpxR* null allele may not affect BFP elaboration in the *bfpF* mutant, since this mutation does not appear to completely block BFP assembly in an otherwise wild-type strain, as evidenced by the reduced but not abolished ability of this strain to undergo LA (Nevesinjac and Raivio, 2005).

**Fig. 9:**
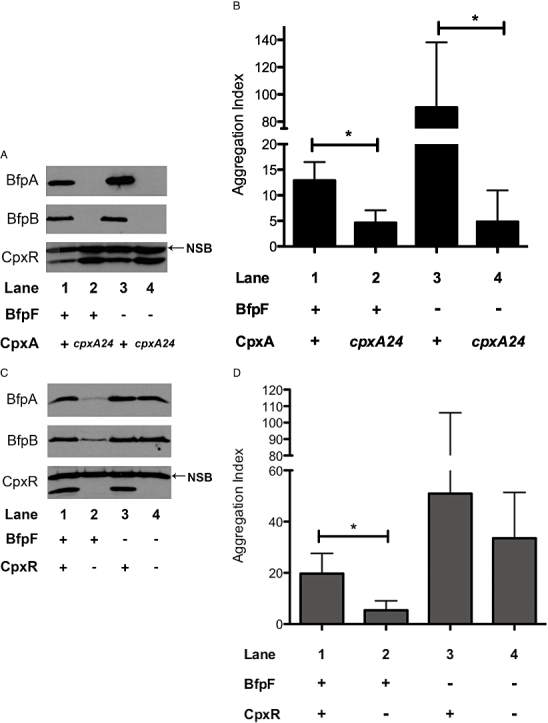
The *cpxA24* mutation, but not the *cpxR*::cam mutation, reduces BFP elaboration in pilus retraction-defective EPEC. A and C. Western analysis of bundlin (BfpA), BfpB and CpxR expression in wild-type and mutant EPEC strains. Whole-cell lysates were collected from EPEC grown in DMEM/F12 as described in *Experimental procedures*. Samples were collected from each strain at least two times; one representative blot is shown. Arrows denote non-specific bands (NSB). B and D. Results of autoaggregation assays, which were performed as described in *Experimental procedures*; the overall average and standard deviation resulting from three separate experiments performed in triplicate, are shown. The asterisk (*) denotes a value significantly different from the relevant *cpx*^+^ control strain (*P* < 0.05, unpaired *t*-test). Shown in (A) and (B) are wild-type strain E2348/69 (lane 1), *cpxA24* mutant ALN195 (lane 2), *bfpF* mutant UMD916 (lane 3) and *bfpF cpxA24* double mutant SV75 (lane 4). Shown in (C) and (D) are E2348/69 (lane 1), *cpxR* mutant ALN234 (lane 2), UMD916 (lane 3) and *bfpF cpxR* double mutant SV82 (lane 4).

The *cpxA24* and *cpxR*::cam alleles were introduced into the *bfpF* mutant UMD916 as described in *Experimental procedures*, and the mutations were confirmed by PCR analysis and anti-CpxR Western blotting, which revealed increased CpxR expression in the *cpxA24* mutant and, as expected, a loss of CpxR in the *cpxR*::cam strain (Fig. 9A and C). We then examined BFP synthesis in the resulting double mutants by Western blotting and autoaggregation assays. Introducing the *cpxA24* mutation into UMD916 strongly reduced BFP synthesis. Bundlin and BfpB could not be detected in this strain by Western blotting (Fig. 9A), and its ability to autoaggregate was reduced to a level comparable to that of the *cpxA24* single mutant (Fig. 9B). Interestingly, introducing the *cpxR::cam* null allele into UMD916 did not appreciably decrease BFP expression, as assessed by Western blotting (Fig. 9C), nor did this mutation significantly reduce autoaggregation of UMD916 (*P* > 0.05, unpaired *t*-test) (Fig. 9D). The phenotypes of the *bfpF* double mutants are therefore consistent with those observed in previous experiments, indicating a defect in BFP assembly (by virtue of a defect in expression of the *bfp* genes) in the *cpxA24* mutant, but no assembly defect in the *cpxR* mutant (i.e. BFP can still be made in this mutant, albeit not as well as in wild-type strains). Rather, the decreased autoaggregation and LA of the *cpxR* single mutant (Nevesinjac and Raivio, 2005) can likely be attributed to unstable pili that are prone to retraction.

## Discussion

In this study, we investigated the mechanism by which the Cpx envelope stress response regulates EPEC BFP expression. Previously, we demonstrated that EPEC lacking a functional Cpx pathway, due to mutation of *cpxR*, exhibits reduced expression of bundlin and diminished LA to tissue culture cells (Nevesinjac and Raivio, 2005). In the current study, we found that this decreased BFP synthesis cannot be attributed to reduced transcription of the *bfp* gene cluster in the *cpxR* mutant (Fig. 3). On the other hand, we found that mutating the Cpx-regulated periplasmic proteins DsbA, DegP and CpxP reduced both BFP protein accumulation and BFP-mediated processes like bacterial aggregation (Fig. 1). Therefore, the decreased BFP synthesis in the EPEC *cpxR* mutant can most likely be explained by insufficient expression of one or more factors crucial for the proper folding of BFP components. Importantly, BFP protein expression could not be increased in the *cpxR* mutant simply by overexpressing the disulphide bond oxidoreductase DsbA (Fig. 2). This finding indicates that the BFP expression defect in the *cpxR* mutant is not simply the product of insufficient DsbA, which was previously shown to be necessary for bundlin stability (Zhang and Donnenberg, 1996). The increased piliation of the *cpxR bfpF* double mutant compared with the *cpxR* single mutant (Fig. 9) supports the assertion that the *cpxR* mutant is fully capable of expressing the *bfp* genes and synthesizing all required BFP proteins. However, the *cpxR* mutant likely assembles a structurally defective pilus that is prone to pilus retraction; this pilus instability is most likely the result of decreased expression of folding factors like DsbA, DegP and CpxP. We believe that the adherence defects of the *cpxR* mutant are more likely the result of pilus retraction rather than shedding of BFP into the culture medium, since we have never been able to detect bundlin in *cpxR* culture supernatants by Western blotting nor have we observed any BFP, attached to cells or sheared off, in the *cpxR* mutant by transmission electron microscopy (Nevesinjac and Raivio, 2005; data not shown).

Since our previous work showed that activating the EPEC Cpx response inhibits type III secretion, we also examined the effect of Cpx pathway activation upon BFP expression. Strikingly, we were unable to detect BFP proteins in the EPEC *cpxA24* mutant (Fig. 4), in which the Cpx response is constitutively active due to a mutation in the periplasmic sensing domain of CpxA. A similar, though less severe, BFP repression phenotype was observed when the wild-type Cpx pathway was activated by overexpressing NlpE (Fig. 5). We believe this inhibition of BFP expression is achieved primarily at the transcriptional level. Transcription of *bfpA* was reduced approximately 20-fold in the *cpxA24* mutant (Fig. 6), and introducing the *cpxA24* mutation into a strain in which the *bfp* gene cluster is expressed from an inducible promoter did not impair BFP synthesis (Fig. 7), demonstrating that the Cpx response does not cause any significant post-transcriptional repression of BFP expression. Our hypothesis is also consistent with the observation that BFP expression was restored to the *cpxA24* mutant by overexpressing the *bfp* transcriptional activator PerA (Fig. 8), which raises the possibility that the Cpx response could influence *bfp* transcription through PerA (discussed in more detail below). In contrast to the results obtained with the *bfpF cpxR* double mutant, BFP synthesis was not restored in a *bfpF cpxA24* double mutant (Fig. 9), again suggesting that the *cpxA24* mutant is incapable of BFP synthesis rather than synthesizing a defective pilus. The Cpx pathway therefore appears to mediate both transcriptional and post-transcriptional effects upon the BFP; the transcriptional effects are negative and are evident only when the pathway is activated, as in the *cpxA24* mutant. On the other hand, the post-transcriptional effects, which are likely mediated by interaction between Cpx-regulated periplasmic protein folding factors and BFP protein components, are positive and occur even when the pathway is basally active, such as in wild-type cells. This is the first report that reconciles the ability of the Cpx pathway to act at multiple regulatory levels to mediate both positive and negative effects upon a single cell-surface structure.

### Role of Cpx-regulated protein folding factors in BFP biogenesis

In this work, we confirmed the importance of DsbA in BFP synthesis, as well as implicating two additional Cpx regulon members in this process: DegP and CpxP. DsbA is the major catalyst of disulphide bond formation in the *E. coli* periplasm (Heras *et al*., 2009) and is required for stability of bundlin (Zhang and Donnenberg, 1996). The C-terminal disulphide bond formed by DsbA in bundlin is a conserved feature of all Gram-negative type IV pilin proteins (Craig and Li, 2008), which implies that disulphide bond oxidoreductases may be essential for type IV pilus biogenesis in a variety of pathogens. Recent studies have shown that DsbA homologues are required for proper folding of the OM secretin PilQ, a component of the *Neisseria meningitidis* type IV pilus (Sinha *et al*., 2008), and that EPEC DsbA is required for stability of the OM secretin EscC, which forms part of the T3S complex (Miki *et al*., 2008). Thus, DsbA may also be required for disulphide bond formation in BFP components other than the pilin itself; indeed, all of the BFP proteins we examined were undetectable or noticeably less abundant in the *dsbA* mutant (Fig. 1A)

Although its phenotype was less dramatic than that of the *dsbA* mutant, the EPEC *degP* mutant also expressed reduced levels of BFP proteins (Fig. 1A), which correlated with decreased abilities to autoaggregate (Fig. 1B) and to adhere to epithelial cells. These data are consistent with those obtained by Humphries *et al*. (2010) in the accompanying manuscript, who found that bundlin expression was delayed in a *degP* mutant compared with wild-type EPEC. This defect was attributed to the loss of the chaperone activity of DegP. To our knowledge, this is the first example of a role for DegP as a chaperone in facilitating pilus assembly.

The expression of BFP proteins was also reduced in the *cpxP* mutant (Fig. 1A). The role of CpxP in BFP biogenesis, however, remains unclear. CpxP has two distinct cellular functions – it acts both as a repressor of Cpx pathway activation (Raivio *et al*., 1999) and as an accessory factor to the protease DegP (Isaac *et al*., 2005). Since we also showed that activating the Cpx pathway results in decreased BFP expression (Figs 4 and 5), it is possible that the reduced BFP expression in the *cpxP* mutant is simply the result of the Cpx pathway being more active in this strain. Another possibility is that CpxP promotes the folding of BFP substrate proteins. Although CpxP has not been shown to act as a chaperone, it is known to facilitate proteolysis of misfolded Pap pilins by DegP (Isaac *et al*., 2005). Areas for future research include whether CpxP might also facilitate the chaperone activity of DegP under certain circumstances, and whether CpxP has a role in delivering bundlin to DegP in a similar manner as the PapE pilin.

Interestingly, we observed that the EPEC mutants expressing a reduced level of bundlin, including the *dsbA*, *degP* and *cpxP* mutants, also expressed lower levels of other BFP proteins contained within different cellular compartments (Fig. 1). This observation extends to the protein BfpD, whose cytoplasmic location precludes a direct interaction with periplasmic folding factors. One possible explanation is that reduced bundlin expression or stability results in proteolysis of other BFP components, or perhaps feedback repression of transcription. However, non-polar mutations in *bfpA* have no effect on the stability of other BFP proteins (Ramer *et al*., 2002), nor do they decrease transcription from the *bfpA* promoter (S.L. Vogt and T.L. Raivio, unpubl. obs.). These data support the idea that Cpx-regulated proteins such as DsbA and DegP are required not only for the folding of bundlin, but likely also assist in the folding of additional BFP proteins as well. Most of the BFP proteins are required for the stability of at least one other BFP component, and often several; in fact, mutating the IM scaffolding protein BfpE destabilizes all of the BFP components to some extent (Ramer *et al*., 2002). Since BfpE contains a large C-terminal periplasmic domain (Blank and Donnenberg, 2001), this protein may require Cpx regulon members for proper folding and stability, which could account for destabilization of the entire pilus in the *dsbA*, *degP* and *cpxP* mutants. Alternatively, Cpx-regulated folding factors may play a more indirect role in BFP assembly, by facilitating the folding of another envelope protein that is essential for this process.

### Transcriptional regulation of *bfp* by CpxR

In addition to these post-transcriptional effects mediated by Cpx-regulated folding factors, we also investigated how the Cpx pathway affects *bfp* transcription. Using a *bfpA–lux* reporter, we found that activating the Cpx response results in decreased *bfpA* transcription (Fig. 6). CpxR therefore acts as a transcriptional repressor of the *bfp* operon, through either direct or indirect means. Given this result, we might have expected that *bfpA* transcription would be elevated in the *cpxR* null mutant. In contrast, we found no difference in *bfpA* transcription between wild-type and *cpxR* mutant strains (Fig. 3). These results suggest that the Cpx pathway affects *bfpA* transcription only when there is a high concentration of phosphorylated CpxR in the cell, such as in the *cpxA24* mutant (Raivio and Silhavy, 1997), which could be the result of a low-affinity binding site for CpxR upstream of *bfpA* or one of its regulators.

Although *bfpA* transcription is attuned to numerous environmental parameters (Puente *et al*., 1996), only PerA has thus far been identified as a direct transcriptional regulator of the *bfp* operon (Tobe *et al*., 1996; Ibarra *et al*., 2003). Numerous environmental signals and genetic regulators feed into transcriptional regulation of *perA* (Martínez-Laguna *et al*., 1999; Sperandio *et al*., 1999; Shin *et al*., 2001; Ferreira and Spira, 2008). PerA, in turn, directly activates expression of the *bfp* operon and indirectly activates transcription of type III secretion genes via PerC and Ler (Porter *et al*., 2004). We favour the hypothesis that CpxR also mediates its transcriptional repression of the *bfp* operon at least partially via repression of *perA*. We observed that a *perA–lux* reporter is expressed at lower levels when the Cpx pathway is activated (Fig. 6). We also found that we could restore BFP synthesis in the *cpxA24* mutant by overexpressing *perA* (Fig. 8). Additionally, we could not detect any CpxR consensus binding sequences upstream of *bfpA* either visually or using the online tool Virtual Footprint (Münch *et al*., 2005). Finally, during time-course experiments examining *bfpA–lux* activity when the Cpx response was induced by overexpressing NlpE, we found that repression of the *bfpA–lux* reporter occurred only 3 h or later after inducing NlpE overexpression (data not shown). In contrast, the expression of genes that are known to be directly regulated by CpxR is altered within 30 min of NlpE overexpression (D.M. MacRitchie *et al*., in preparation). The slow kinetics of *bfpA* repression is suggestive of indirect regulation. At this time, we cannot say whether CpxR directly represses transcription of *perA*, represses transcription of *perA* through another regulator, or perhaps affects *bfpA* through another, yet to be identified regulator, thereby possibly inducing negative feedback on *perA* expression. Direct repression of *perA* seems least likely because, as with the *bfpA* promoter, we could not find a CpxR consensus sequence upstream of *perA*, and the kinetics of *perA* repression after NlpE overexpression are comparably slow. Another intriguing possibility is that CpxR regulates PerA post-transcriptionally, for example by upregulating a cytoplasmic protease that degrades PerA, thereby also reducing *perA* transcription by preventing PerA autoactivation (Martínez-Laguna *et al*., 1999). Further studies will hopefully elucidate the molecular mechanism(s) by which the Cpx pathway affects this important regulator of EPEC virulence.

### Regulation of pilus expression by the Cpx response

In addition to its role in regulating BFP expression, the Cpx response also modulates the expression of several other types of pili in *E. coli* strains. The Cpx response represses transcription of the structural and regulatory genes for synthesis of curli, adhesive structures involved in surface attachment and biofilm formation (Barnhart and Chapman, 2006), via direct binding of CpxR∼P to the promoter regions of the relevant genes (Dorel *et al*., 1999; Prigent-Combaret *et al*., 2001; Jubelin *et al*., 2005). Additionally, when the Cpx pathway is activated, transcription of the *pap* structural genes, encoding the chaperone-usher-type Pap pili of uropathogenic *E. coli*, is reduced through a mechanism involving the inhibition of Lrp-mediated phase variation (Hernday *et al*., 2004). Conversely, and parallel to results obtained here with BFP, *cpxR* mutations decrease Pap pilus elaboration even when the *pap* genes are expressed from an inducible promoter, an effect that has been attributed to diminished expression of periplasmic protein folding and degrading factors (Hung *et al*., 2001). Finally, similarly to curli and the Pap pilus, Cpx-mediated inhibition of elaboration of the conjugal F-pilus also occurs by transcriptional repression of the *tra* operon encoding F-pilus structural components (Sambucetti *et al*., 1982). In this case, however, the action of CpxR is indirect. Activation of the Cpx pathway results in upregulation of the cytoplasmic protease/chaperone pair HslVU (Lau-Wong *et al*., 2008), leading to degradation of the *tra* operon activator TraJ (Gubbins *et al*., 2002; Lau-Wong *et al*., 2008). Therefore, Cpx-regulated protein folding and degrading factors seem to promote the elaboration of several pilus types, while activating the Cpx response represses transcription of pili-encoding genes. This finding may point to the importance of preventing pilus component accumulation in the periplasm when the cell is attempting to recover from a period of envelope stress and folding factors required for pilus assembly would be limiting.

### Significance of the Cpx pathway to EPEC pathogenesis

The results presented in this report demonstrate that the activated Cpx response strongly inhibits the expression of BFP, which are believed to be a major adhesin responsible for EPEC early adherence (Cleary *et al*., 2004; Hyland *et al*., 2008). These findings raise the question of whether induction of the Cpx response is likely to occur during EPEC colonization of the human intestine. In the accompanying manuscript, Humphries *et al*. (2010) have demonstrated that the Cpx response is actually downregulated by BFP retraction induced by the receptor analogue LacNAc-BSA. This observation suggests that the Cpx response is unlikely to be triggered by the binding of EPEC to the host epithelium. Perhaps activation of the Cpx pathway is more likely to occur during the transmission phase of the EPEC life cycle, when bacteria can be faced with a variety of stresses in the abiotic environment and when expression of pili may be disadvantageous. However, our results predict that a basal level of Cpx pathway activity is likely important during the early stages of infection, since BFP are not fully expressed and appear to be prone to retraction in the absence of CpxR.

This work, in combination with that of MacRitchie *et al*. (2008b), shows that activating the Cpx response inhibits the expression of two major EPEC virulence determinants, BFP and the type III secretion system. At least part of this inhibitory effect may be the result of decreased expression of *perA* (Fig. 6), the master regulator of virulence in EPEC. Activation of the Cpx response appears to also repress virulence processes in the related enteric pathogen *Salmonella enterica* serovar Typhimurium (Humphreys *et al*., 2004). As such, a chemical inducer of the Cpx envelope stress response could represent a valuable therapeutic tool, potentially capable of preventing intestinal colonization by numerous human pathogens.

## Experimental procedures

### Bacterial strains and growth conditions

All bacterial strains and plasmids used in the course of this study are listed in Table 1. EPEC strains were routinely cultured in LB broth containing the appropriate antibiotics at 37°C with shaking at 225 r.p.m. Strains harbouring *degP* or *cpxA24* mutations were routinely cultured at 30°C with shaking, except when performing assays to detect BFP expression (whole-cell lysates and luminescence assays). Under these circumstances, all strains were grown at 37°C to induce maximal BFP expression. When required, isopropyl-β-d-thiogalactopyranoside (IPTG) (Invitrogen) was added to a concentration of 0.1 mM, unless otherwise indicated. Antibiotics (all from Sigma) were added as necessary to the following concentrations: amikacin (Amk), 3 µg ml*^-^*^1^; ampicillin (Amp), 100 µg ml*^-^*^1^; chloramphenicol (Cam), 25 µg ml*^-^*^1^; and kanamycin (Kan), 50 µg ml*^-^*^1^.

**Table 1:** Bacterial strains and plasmids used in this study.

Strain or plasmid	Description	Source or reference
Bacterial strains		
E2348/69	Prototypical EPEC O127:H6 strain	Levine *et al*. (1978)
JPN15	Spontaneous pEAF-cured derivative of E2348/69	Jerse *et al*. (1990)
ALN88	E2348/69 *cpxR*::Kan^R^	Nevesinjac and Raivio (2005)
ALN188	E2348/69 *degP*::Kan^R^	D.M. MacRitchie *et al*. (in preparation)
ALN190	E2348/69 *ppiA*::Kan^R^	D.M. MacRitchie *et al*. (in preparation)
ALN194	E2348/69 *cpxP*::Kan^R^	D.M. MacRitchie *et al*. (in preparation
ALN195	E2348/69 *cpxA24* (Amk^R^)	MacRitchie *et al*. (2008b)
ALN234	E2348/69 *cpxR*::Cam^R^	MacRitchie *et al*. (2008b)
TR1121	E2348/69 *dsbA*::Kan^R^	D.M. MacRitchie *et al*. (in preparation)
SV76	JPN15 *cpxA24* (pKDS302) (Amk^R^ Amp^R^)	This study
UMD916	E2348/69 *bfpF*::Kan^R^	Anantha *et al*. (1998)
SV75	UMD916 *cpxA24* (Kan^R^ Amk^R^)	This study
SV82	UMD916 *cpxR*::Cam^R^ (Kan^R^)	This study
Plasmids		
p*trc*99A	High copy-number expression vector with IPTG-inducible promoter (Amp^R^)	Pharmacia
pDsbA	p*trc*99A-based *dsbA* overexpression vector (Amp^R^)	Buelow and Raivio (2005)
pJW22	*perA* promoter cloned into *luxCDABE* reporter vector pJW15 (Kan^R^)	MacRitchie *et al*. (2008b); this study
pJW23	*bfpA* promoter cloned into *luxCDABE* reporter vector pJW15 (Kan^R^)	MacRitchie *et al*. (2008b); this study
pCA24N	High copy-number expression vector with IPTG-inducible promoter (Cam^R^)	Kitagawa *et al*. (2005)
pCA-*nlpE*	pCA24N-based *nlpE* overexpression vector (Cam^R^)	Kitagawa *et al*. (2005)
pKDS302	p*trc*99A containing the *bfpA-L* gene cluster expressed from an IPTG-inducible promoter (Amp^R^)	Stone *et al*. (1996)
pMPM-K3	Low copy-number cloning vector derived from pACYC184 and pBluescript (Kan^R^)	Mayer (1995)
pCS-A	pMPM-K3-derived *perA* overexpression plasmid (Kan^R^)	Martínez-Laguna *et al*. (1999)

### Strain construction

To construct strain SV76, plasmid pKDS302, which contains the fourteen-gene *bfp* cluster expressed from P*trc* (Stone *et al*., 1996), was electroporated into strain JPN15, a derivative of E2348/69 that was spontaneously cured of the *bfp*-encoding EAF plasmid (Jerse *et al*., 1990). The suicide plasmid pRE112*cpxA24* was subsequently transferred into the transformed strain by biparental mating as previously described (MacRitchie *et al*., 2008b). The presence of the *cpxA24* mutation in this strain was verified by PCR amplification of *cpxA*, showing a deletion of approximately 100 bp, and by confirmation of growth on media containing 3 µg ml*^-^*^1^ amikacin. Strain SV75 was constructed in a similar manner, except that pRE112*cpxA24* was conjugated into UMD916.

The *bfpFcpxR*::cam double mutant strain SV82 was constructed by conjugating the suicide plasmid pRE118*cpxR*::cam (MacRitchie *et al*., 2008b) into strain UMD916 and selecting for chloramphenicol- and sucrose-resistant colonies. Presence of the *cpxR* insertion mutation was verified by PCR analysis (not shown) and Western blotting (Fig. 9C).

### Construction of *bfpA–lux* and *perA–lux* reporter plasmids

To construct the *bfpA–lux* reporter plasmid pJW23, the *bfpA* promoter region was amplified from E2348/69 using the primers ProBFPA-*Eco*RI (5′-GTGAATTCTGCAGGGGAATAATGTTGTTC-3′) and ProBFPA-*Kpn*I (5′-GGGGTACCCCAAGCACCATTGCAGATT-3′) (underlining denotes restriction enzyme tag). The resulting PCR product was purified and digested with the restriction enzymes *Eco*RI and *Kpn*I, then ligated into the multiple cloning site upstream of the promoterless *luxCDABE* operon in pJW15 (MacRitchie *et al*., 2008b), using standard techniques. A similar procedure was followed to generate the *perA–lux* reporter plasmid pJW22, except using the primers proPER-L (5′-CGGAATTCTACTCACTTAGCCGCGTGTC-3′) and proPER-R2 (5′-GGGGTACCTTAACAATAACGCTAAATTCTCCTC-3′) and the restriction enzymes EcoRI and BamHI.

### Western blot analysis

Whole-cell lysates for Western blot analysis were generally prepared by subculturing EPEC strains in DMEM: Nutrient Mixture F-12 (DMEM/F12, Gibco) containing 0.1 M Tris (pH 7.4) as previously described (Nevesinjac and Raivio, 2005), with cultures grown to an OD_600_ of 0.5–0.6. The strains carrying pKDS302 or its vector control p*trc*99A were subcultured in LB containing 1 mM IPTG. Electrophoresis and Western blotting were performed as previously described (Raivio *et al*., 1999). Blots were incubated with primary antisera at the following concentrations: anti-bundlin (Fernandes *et al*., 2007), 1:5000; anti-BfpB (Daniel *et al*., 2006), 1:10 000; anti-BfpC (Crowther *et al*., 2004), 1:1000; anti-BfpD, 1:2000; or anti-MBP-CpxR, 1:10 000 (Raivio *et al*., 1999). BfpD antiserum was raised in guinea pigs against BfpD protein, purified as described (Crowther *et al*., 2004). The secondary anti-rabbit (or for BfpD anti-guinea pig) immunoglobulin G-alkaline phosphatase conjugates (Sigma) were used at a concentration of 1:25 000. Blots were developed with the chemiluminescent Immun-Star AP Substrate Pack (Bio-Rad) according to manufacturer's directions.

### Autoaggregation assay

Autoaggregation assays were performed as previously described (Nevesinjac and Raivio, 2005). Most EPEC strains were subcultured in DMEM/F12 containing 0.1 M Tris (pH 7.4) for these assays, except those strains carrying pKDS302 or p*trc*99A, which were subcultured in LB containing 1 mM IPTG. Assays were performed in triplicate at least two times.

### Localized adherence assay

The ability of EPEC strains to exhibit LA to HEp-2 tissue culture cells was assessed as previously described (Vanmaele and Armstrong, 1997), with bacteria incubated on host cells for 1 h. Experiments were performed two times in triplicate.

### Luminescence assay

Activity of the *bfpA–lux* and *perA–lux* reporters was assessed as previously described (MacRitchie *et al*., 2008b), with EPEC strains subcultured in DMEM/F12 containing 0.1 M Tris (pH 7.4) at 37°C with aeration. Data presented here represent growth for 4 h post subculture unless otherwise stated. Assays were performed at least two times in quintuplicate.
